# The Seven Ages of Man

**Published:** 1888-10-13

**Authors:** 


### The Doctor's Pulpit.

### THE SEVEN AGES OF MAN.

### II.—The Schoolboy.

"The schoolboy, with his shining morning face."

A very popular picture in country cottages represents a village urchin, tied hand and foot in a wheelbarrow, and a stout labourer, presumably his indignant parent, wheeling the refractory imp compulsorily to school. Companion pictures may now and again be seen. One of these displays the master as a stern tyrant, spectacles on nose, surveying his subjects from the elevation of a painted wooden desk, with eyes and look that strike terror to the breast of the stoutest birdnester, and make the more timid youngsters tremble as they fashion their painful pot-hooks. The other shows a milder type of pedagogue. He, absorbed in some profound question in rule of three or vulgar fractions, is entirely oblivious of the behaviour going on around. Master Robinson, aged ten, is stealing a kiss from Miss Green, aged eight, who returns the salute by an energetic use of her finger-nails. The pupils of still tenderer years look up from their inky slates, and glance from the culprits to the master and back again with alarmed amusement.

But whatever kind of picture it is that represents school and schoolmaster, in every case the latter is a "most prodigiously great man." Whether it be the learned Dr. Busby marching up and down the school before the king himself, and overwhelming the boys with his superior majesty, or Goldsmith's miracle of village genius, of whom we know that, after years of familiarity—

Still the wonder grew,
That one small head could carry all he knew—

even to this day, the schoolmaster, in whatever form, is to the wondering pupil a portentous, a lofty, and an unapproachable personality. The present writer well remembers the schoolmaster of his early days, and the grave "Doctor" of his later schoolboy period. No University Professor of worldwide reputation was ever so great in his eyes as either of those. Of true admiration, of willing subjection to acknowledged superiority, of unquestioning belief in universal infallibility, nobody receives so full, unmeasured, and ungrudging a share as the reasonable, kind, and capable schoolmaster.

This picture of the schoolmaster evolves for us perhaps the most correct idea we can have of the schoolboy. He is first and chiefly a crude and wondering student of human life as seen in his teacher. Such an aspect of a young human being deserves careful, and, if possible, unprejudiced consideration on the part of all who look to the future of individuals and of the commonwealth. The schoolboy is in the condition of more or less soft wax, and all kinds of temporary and permanent impressions may be made upon his physical, intellectual, and moral individuality. What is the boy's body to become; what his mind; what his character? We place the body first in order because nature has set us the example; and, curiously enough, St. Paul pronounces definitely on this topic. To all men it is evident that the child's physical organism is first in the order of precedence; first the bodily, then the intellectual, then the moral faculties appear. "That which is first is natural," says St. Paul, "afterwards that which is spiritual."

As a mere matter of looks it is in the highest degree desirable that "the human form divine" shall always approach as nearly as possible to the typically perfect standard. Few people can see an adult man, shorn of half his length by a crooked spine, disabled in his walk by a diseased hip, or pitted deeply in the skin of his face by small-pox, without a feeling of compassion and regret. In almost every way, whether in business, or in competition with other men in the choice of a wife, or in his relations to society generally, the deformed and disfigured man is at a disadvantage. He is unfairly handicapped throughout the whole of life by his deformity. That produces in him very often a sense of injury and wrong, thereby tending to mar the wholesomeness and completeness of his character. In the spectator it gives rise to a feeling of superiority which ought not to exist. He unconsciously looks down upon his brother man from an elevation which would be no elevation but for the misfortune which has befallen his unhappy fellow. What every man, who is worth the name, desires is that all men shall at least start fairly in the race of life, and that only he shall win the highest prize whose speed and endurance are found the greatest. The schoolboy age is a formative period, and everything that is possible should be done by parents and schoolmasters to secure for the future man that size, beauty, health, and vigour which constitute the principal part of the capital with which the majority of men are endowed in the struggle for existence. The least every child born into the world can ask of his fellows is that his body shall have a fair chance of healthy and beautiful development until such time as he is freed from all control but his own. When that adult period of life arrives, he alone is responsible for his future strength and health.

From the body to the mind is a natural progression. The comparison with soft wax is perhaps more applicable in the case of the mind than even of the body. It is a curious speculation what kind of men and women the next generation would be if they found themselves from the dawn of consciousness in a world of morally, intellectually, and physically perfect human beings. It need not be imagined that the whole generation would be equally perfect. But that they would be such a company as the world has not yet seen cannot be for a moment doubted. The influence of older persons upon younger, of parents upon children, of teachers upon pupils, is immeasurable. Thousands of children and young people of the noblest disposition, of the most generous temper, and of the finest intellectual aptitudes, grow up narrow-souled, prejudiced, mean-tempered, and mentally incapable because of the steady and constant pressure of the environment in which they find themselves. On the other hand, not a few who are born mentally crooked and defective are made comparatively straight and human by careful training. We have often protested in these pages against the wicked lack of common sense which frequently prevails among those who are responsible for the methods in use in the Board and other lower-class schools. It is necessary to say, and say again, that hungry children are slow and feeble learners. They want to be fed. All children are incapable of sustained application to books for more than four or five hours daily. To keep them in full work for five hours during the day, and then to give them a couple of hours' home work for the evening, is the very madness of self-satisfied ignorance and incompetence. We shall never be content until the wise and experienced medical man has his recognised official position, and exercises a constant supervision over all those who are perforce committed to the care of State educators. If the State insists upon educating the people, we should at least see to it that it does not injure the brain and health in its efforts to improve the mind. The mental training of the young stands second to no occupation. Those who are entrusted with it should be equal, if not superior in education and character to either the clergyman, the lawyer, or the doctor.

The schoolboy has a conscience as well as a body and reasoning faculties. The conscience wisely handled is the best pair of reins a parent or teacher can have in the management and direction of childhood. The main points with regard to the conscience of the boy and girl period are two. Justice should always be done to the child, and in his presence; he will then always feel that justice may be rightly demanded at his own hands. There is nothing more dangerous and more deadly to the moral faculty in children than tampering with conscience. At this early period of life it is quick and sensitive in a high degree: often much quicker and more sensitive than in later life. It is at this period that the careless or unconscientious parent or teacher may stupefy and destroy with the deadly narcotic of his own example and influence the noblest part of the future man. A sound mind in a sound body should be the aim of all who have the control of boys and girls; but still more should every effort be made to strengthen and develop an honourable and virtuous character. Nature herself shows us the order and relative value of those characteristics which distinguish man. The body is, so to speak, the root and stem, the mind is the flower and perfume, but the soul and character are the perfect and ripened fruit. Let every parent and teacher who has such human trees in his garden see to it that they are both healthy, beautiful, and fruitful.

